# Supplementary material for: Template-Free Manufacturing of Defined Structure and Size Polymeric Microparticles
Source: Nanomaterials (Basel). 2023 Nov 20;13(22):2976. doi: 10.3390/nano13222976 (PMC10674349; doi:10.3390/nano13222976)
Supplement: Supplementary file 1 [file nanomaterials-13-02976-s001.zip › nanomaterials-2680791-supplementary.pdf]

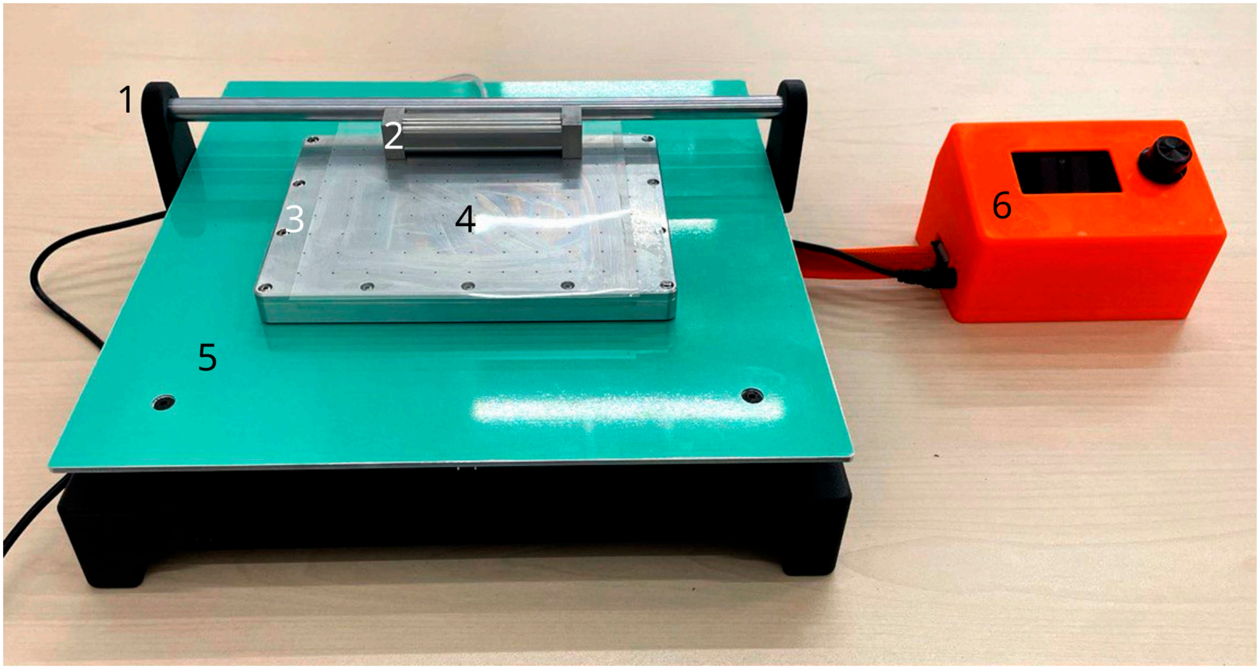

Figure S1. Home-build film applicator unit.

1. Moving bar
2. Baker film applicator with discrete gaps
3. Vacuum table for thin substrate fixation
4. FEP substrate
5. Base table
6. Control unit
